# Green Innovation Practices and Its Impacts on Environmental and Organizational Performance

**DOI:** 10.3389/fpsyg.2020.553625

**Published:** 2021-01-18

**Authors:** Haijun Wang, Muhammad Aamir Shafique Khan, Farooq Anwar, Fakhar Shahzad, Daniel Adu, Majid Murad

**Affiliations:** ^1^School of Management, Jiangsu University, Zhenjiang, China; ^2^Lahore Business School, University of Lahore, Lahore, Pakistan

**Keywords:** innovation orientation, competitor pressure, employees’ conduct, green innovation, environmental performance, organizational performance

## Abstract

This study aims to investigate the impact of stakeholders’ views on the practices of green innovation (GI), consequent effect on environmental and organizational performance (OP), and moderating influence of innovation orientation. A quantitative method was employed for the sample size of 515 responses. To accumulate the data from the respondents, convenient random sampling was used. Data were collected from manufacturing and services firms through a field survey by using a closed-ended questionnaire based in the Punjab province of Pakistan. The analysis was done using the structural equation model of the partial least square analysis method. Our findings proved a positive and significant link between stakeholders’ views on GI practices. A significant association has been found between GI practices and environmental and OP. The moderating effect was found to be negative but statistically significant. This research offers numerous contributions and provides decision-making insinuations.

## Introduction

Resource limitations and environmental concerns have made sustainable operations of assets and environmental pollution one of the major global issues. The economy’s overall development may not go “hand in hand” with the reduction of pollution and sustainable management of resources (Wang and Song, 2014). Building a sense of balance among high resource consumption and development of economy relics is a constant challenge that forces organizations to run-through eco-friendly professional deeds having high economic worth (Chan et al., 2012). Many organizations are forced to adopt activities that generate and increase economic value (Porter and Kramer, 2019).

The excessive use of non-renewable resources prompted by speedy economic development has hurt the atmosphere and elevated various environmental worries (Atlin and Gibson, 2017). To preserve energy and lessen emissions of carbon, numerous countries have established agencies and regulations for environmental sustainability and its protections; examples comprise limitations on “chlorofluorocarbons, the sustainable development announcements of the Johannesburg world summit,” and limits on the usage of few hazardous materials “electrical and electronic equipment requirements, the European Union’s Restriction of Hazardous Substances Directive” (Weng et al., 2015, p. 4998). Such impositions of rule and regulations have drawn the attention of environmental supervisors (Zhu and Sarkis, 2004; Claver et al., 2007); they also have the same outcome in varying the management and competition practices between the organizations (Feng and Chen, 2018). To adhere to the new eco-friendly regulations, to have a positive branding image (Chen, 2008a; Hillestad et al., 2010), to improve their firms’ performance and to have a competitive advantage (Claver et al., 2007; Rusinko, 2007), organizations have had to accept eco-friendly practices (Afridi et al., 2020).

Numerous investigations examined factors altering green innovations (GI) practices, such as environmental regulations, ethics, legal systems, and supply chain (Feng and Chen, 2018; Gao et al., 2018; El-Kassar and Singh, 2019; Seman et al., 2019). Studies have also examined an increase in awareness, the general public, and stakeholder pressure linked to green environmental issues (Foo, 2018). Moreover, literature provides evidence of optimized pressure from society, customers, and government bodies to practice GI. However, the literature lacks findings on the relationship of stakeholders’ pressure [competitor’s pressure, government pressure, and employee conduct (EC)] about GI practices. The manufacturing sector faces higher stakeholder pressure due to possibly the highest waste-producing sector (Chen, 2008b; Chang, 2011). The single industry was studied for GI practices (Cordano et al., 2010; Lin and Ho, 2011). This study fills the gap in investigating these constructs in the manufacturing and service industries to enrich existing GI practices and stakeholder pressure literature. Moreover, stakeholder pressure (customer) was examined for GI in third party logistic firms (Chu et al., 2019), as well as in express companies (Zhang et al., 2020), and in manufacturing firms (Song et al., 2020). Those three studies were conducted in China’s context, which highlights the issue of conducting and focusing on the stakeholder pressure in the manufacturing and service industries of Pakistan being a developing economy in the initial stages of GI practices adoption (Shahzad M. et al., 2020).

“Go-green” is an initiative mainly employed by firms to deal with eco-friendly problems. Approaches to attain green abilities and emerging eco-friendly practices have focused on attention and discussion in the management sciences’ discipline over the years (Ullah, 2017). To ease the acceptance of GI, firms must consider the significant factors and precursors in their business entities (Arfi et al., 2018). These comprise apprehensions of consumers (Zhu et al., 2017), preferences of professionals and owners (Huang et al., 2009), competency of suppliers and partners (Chiou et al., 2011), government regulating authorities and their regulations (Kammerer, 2009), and the environmental, technological, and organizational factors of GI practices (Lin and Ho, 2011). Green technologies consist of GI practices (e.g., green product, process, managerial, and marketing innovation) and the execution of green human resource management practices (e.g., green training and development, administrative support and culture, recruitment and selection, compensation, and benefits). GI is a significant strategic enabler to acquire justifiable development, as it practices energy-saving, environment-protecting, waste-recycling, and pollution-preventing methods (Albort-Morant et al., 2018). Furthermore, GI can be divided into green product, green marketing, green processes, and green management that are intended for eco-friendly environment, decreasing consumption of energy and increasing efficient use of the resource, control over pollution emission, and waste recycling, improving the performance of the organization and providing the pollution-free environment to society at large scale (Seman et al., 2019).

Previous studies have witnessed some proofs of the impacts of numerous drivers such as corporate environmental ethics (El-Kassar and Singh, 2019), environmental regulations (Feng and Chen, 2018), the legal system (Gao et al., 2018), and green supply chain management practices (Seman et al., 2019) on GI practices. To date, some systematic and comprehensive investigations of the precursors and factors of GI have been performed. Foo (2018) proposed that the increase in awareness and pressure from the stakeholders and the general public have necessitated organizations to be more transparent in facing and handling green environmental issues of their supply base execution. Hence, it is critical to focus on stakeholders’ views in an organization on establishing and sustaining GI abilities and practices. Then executives of organizations are involved in examining the essential factors necessary for creating GI practices. Are there pressures from established institutions’ regulations and competitor’s critical factors of GI? How should firms have dealt with the concerns of both internal and external stakeholders?

Furthermore, previous studies have concentrated on the manufacturing sector as it is one of the most critical waste producers that upset the balance of an environment. With rising trepidations on global pollution, this industry is facing increasing pressures from customers, society, and governing agencies to save energy, resources, protect the eco-friendly environment and maintain its sustainability (Chen, 2008b; Chang, 2011) or on a single industry (e.g., Cordano et al., 2010; Lin and Ho, 2011). It would be beneficial to offer an all-purpose model to investigate issues about GI for both the service and manufacturing firms. Therefore, in this study, we borrowed help from the “stakeholder theory” (Freeman, 2010) to aid in our investigation methodology. This theory has been utilized to get a comprehensive view of a particular organization to examine stakeholders’ influence (participants) on GI practices. To answer the stakeholders’ pressure, organizations should focus on an overall strategic plan that involves and satisfies both internal and external stakeholder groups (Bryson, 2018).

## Review of Literature

### Stakeholder View (SV)

The word “stakeholders” was initially used by the “Stanford Research Institute” in 1963 and was defined as “those groups without whose support the organization would cease to exist” (Friedman and Miles, 2006). While this concept was first brought into a “strategic discipline” in 1984 by Freeman (1984), stakeholders were not only separate from shareholders but also involved in the decision-making process (Donaldson and Preston, 1995; Mitchell et al., 1997). In an academic view, the “stakeholder theory” holds a unique perspective for the organizations and offers a diverse description of a firm’s structure and everyday actions (Sulkowski et al., 2018). The stakeholder theory, founded on four indispensable grounds (Jones and Wicks, 1999), first suggests that organizations have associations with several procedures, all of which are upset or pretentious by their results (Laplume et al., 2008; Co and Barro, 2009). Second, such links are recognized in the firms’ procedures and results and their stakeholders’ firms’ views.

Third, stakeholders’ inherent value, and comforts cannot be permitted to override the safeties of others (Clarkson, 1995; Co and Barro, 2009). Fourth, the decision making of the organizations is the central point (Alrowwad et al., 2017). Stakeholder theory has been accepted for numerous ecological scholarships in that it has been active in persuading both company environmental sensitivity (Crane and Livesey, 2017) and environmental policies (Salem et al., 2018). Although the outcomes have been mixed, and the stakeholders’ views on ecological management have been unpredictable. For example, Jaaffar and Amran (2017) found that the organizations’ board of directors is involved in deciding eco-friendly strategies and policies while small business entities and proprietors decide GI (Huang et al., 2009). In addition, in manufacturing organizations in Germany, stakeholders have affected the firms’ selections concerning ecological response forms (Murillo-Luna et al., 2008), and they were confidently related with unproved GI (Wagner, 2007); in contrast, the association among eco-friendly policies and stakeholders’ administration was not perfect in Belgian organizations (Buysse and Verbeke, 2003). The review paper by Seman et al. (2018) concludes that the stakeholders’ views have a more considerable influence on GI practices.

### Green Innovation (GI)

Works of GI are commonly divided into two types. The first describes GI as a firm’s abilities (Gluch et al., 2009), whereas the second defines GI as an organization’s environmental practices (Lin and Ho, 2008; Ho et al., 2009). When it comes to organizational practices, GI is described as “the hardware or software innovation related to green products or processes” (Song and Yu, 2018); it is proposed that GI comprises management practices and technological advancements that expand the environmental and organizational performance (OP) and provide a competitive edge to the firms (Rennings, 2000). Other researchers recommend that GI consists of unique or altered systems, processes, products, and practices that provide an advantage to the environment and subsidize firms’ sustainability (Xie et al., 2019).

A recent study expresses GI as “the new or modified products and processes, including technology, managerial, and organizational innovations, which helps to sustain the surrounding environment” (Ilvitskaya and Prihodko, 2018). Moreover, GI may refer to “a creative initiative that reduces negative environmental impacts or that yields environmental benefits as it creates value in the market” (Chen et al., 2006). GI is divided into two kinds, such as “green product innovations” (providing new green products to consumers) and “green process inventions” or “greening” business procedures (Tang et al., 2018). Furthermore, due to the growing customer-centered apprehensions concerning environmental protection, ecological management has become a critical part of many firms’ strategic policies and tactical plans (Chiou et al., 2011; Khan et al., 2019).

Regulations related to an environment may lead toward a “win-win situation” (Chan et al., 2018) since they can perform dual tasks, increase profits and lessen pollution; It is proposed that GI should be categorized distinctively from other innovative maneuvers since it harvests not only a spillover consequence for exploration and expansion efforts but also optimistic external possessions such as enlargements in the atmosphere (Kammerer, 2009). A study by Feng et al. (2018) on the Chinese industry’s manufacturing firms has shown that internal and external environmental orientation is significantly associated with GI practices. The utilization of GI practices inside and outside the firms’ restrictions are vital for impacting both economic and ecological performance goals (Khan and Qianli, 2017; Saeed et al., 2018). Moreover, Lee et al. (2018) found that stakeholders’ pressure, organizational support, and societal expectations were significant factors for the motivation to adopt GI practices and corporate environmental responsibility (Shahzad F. et al., 2020). Moreover, the study of Fernando et al. (2019) showed that GI, regulation, supplier intervention, and technology have a strong influence on sustainable performance mediated by service innovation capabilities. The study by Famiyeh et al. (2018) also supported eco-friendly practices, showing that environmental management practices have direct and indirect positive effects on environmental performance. Xie et al. (2019) used green product innovation as a moderator for the green process innovation and OP, but the study did not find the supported results.

## Proposed Framework and Hypothesis Development

### Proposed Framework

**FIGURE 1:**
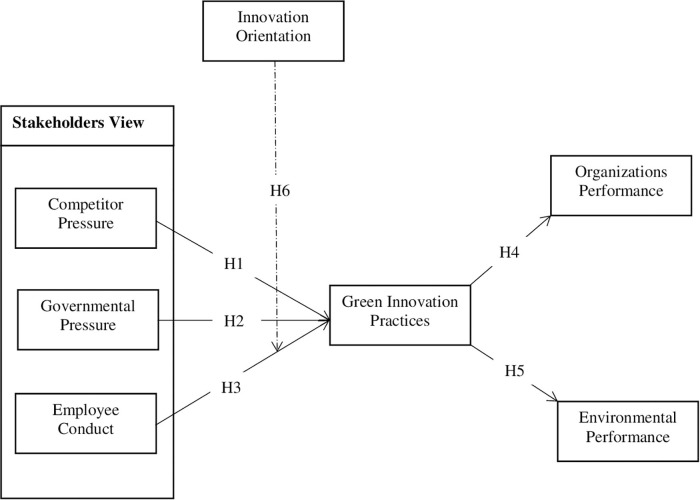
Conceptual model of the study.

This study involves the three dimensions of stakeholders’ view (e.g., competitor pressure, government pressure, and employees conduct) as independent variables. Organizational and environmental performance are used as dependent variables. Moreover, GI practices (e.g., green product and green process) are used as mediators, and the moderating role is performed by innovation orientation (IO). A total of six hypotheses have been suggested and showed in Figure 1.

### Hypothesis Development

We followed “Freeman’s stakeholder framework” (Freeman, 2010). We used three stakeholders’ dimensions to view the government’s and competitors’ pressure as external and employees’ conduct as internal stakeholders. However, there are various other dimensions, such as customer, community, and supplier pressure. This study also treats both aspects of stakeholder’s views as factors that are employing pressure on the organizations and motivating the firms to improve environmental practices. Identifying eco-friendly business practices are becoming critical elements as organizations are confronted with “both internal and external forces/pressures from environmental agencies, governmental regulations, stakeholders, competitors, customers and employees” (Wang and Song, 2014). Singh and El-Kassar (2018) conclude that the stakeholders’ view (e.g., pressure by the government, competitors, employees, customers, society, and suppliers, respectively) positively influences the GI practices.

#### Competitors Pressure (CP)

Organizations generally act in response to the movements of rivals and the operating industry. When competitors accept or implement new eco-friendly practices, organizations in the same sector will feel overstretched to reconfigure the structures and policies (Durand and Georgallis, 2018). In short, organizations need to be attentive to their competitor’s products/services, actions, and norms and regulations of the industry they are part of so that their innovation abilities are similar to others in the industry. For instance, organizations must be conscious of new energy-saving, waste-recycling, pollution-preventing methods, and changes in processes used for the implementation and paraphernalia that are accessible in the market. They are required to have an eye on the methods their competitors have adopted to lessen energy costs while restructuring process and reconfiguring their manufacturing facilities to overtake/perform equivalent to/better than their rivals. Thus, to endure competitive spots, organizations may emulate competitors’ environmental practices and actions, especially the front-runners in their industries (Abrahamson and Rosenkopf, 1993). Singh and El-Kassar (2018) found a positive relationship between stakeholders’ views and GI practices. Furthermore, a study on 442 Chinese firms also confirmed that competitors’ pressure provides organizations with more significant incentives to adopt GI practices (Cai and Li, 2018). In another study (Yu, 2019), the results revealed that formal and informal environmental regulation and pressures have strong influences on food-making companies’ GI activities. Thus, hypothesis 1 is established:

H_1_: Competitor’s pressure has a significant impact on GI practices.

#### Governmental Pressures (GP)

Various scholarships have explored the association among regulatory rules and environmental practices and have proposed that governmental pressures (GP) is a crucial factor of external stakeholders (He et al., 2018). Variations in regulations and implementation of these changes by the government disturb organizational activities concerning environmental management (Yakubu, 2017). In particular, to compete internationally, organizations must keep an eye on both international and national laws to overcome any obstacle. The consistency of the rules and organizations’ insights into the severity of the regulations will define the degree to which firms essentially execute environmental prevention practices (Bernauer et al., 2007). The appropriate governance mechanisms and structural design can successfully manage and supervise the association between nature and mankind (Famiyeh et al., 2018). Moreover, Tirabeni et al. (2019) showed that organizations are reevaluating their manufacturing processes in response to “societal and governmental” pressures concerned with eco-friendly well-being. Furthermore, the degree to which the government enforces/supports the regulations has a substantial influence on the firms’ environmental strategies (Lindell and Karagozoglu, 2001; Zeng et al., 2011), creating a significant task to examine. A study by Zhang et al. (2019) on 224 firms of the manufacturing industry found that institutional pressure significantly affects green supply chain management practices and business performance. In a study by Huang et al. (2016), results show that customer and regulatory pressure encourage green response and increase performance. A survey by Fernando and Wah (2017), based on Malaysian firms, concluded that compliance with government regulations impacts environmental performance. Hence, we suggest hypothesis 2:

H_2_: Governmental pressure has a significant impact on GI practices.

#### Employee Conduct (EC)

Top management identifies the significance of environmental prevention and their responsibility to impact strategic planning and long-term goals related to environmental management. Steady appreciation and consideration of environmental drivers by the management should produce improved innovation and overall performance. Additionally, an organization’s future direction of ecological practices/activities mostly depends on the top management’s commitment toward the utilization of green practices and whether the executives can motivate employees to actively contribute to environmental management (Tang et al., 2018). The same circumstances exist between employees. In a business, workforces are often the originators of environmental practices (Daily and Huang, 2001). Organizations will strain to achieve ecological goals if the personnel/workforce do not contribute to their policies and strategies (Zhu et al., 2008). Thus, firms must arrange and offer workshops and training on environmental concerns, include suitable employees, and improve their obligation to eco-friendly practices (Reinhardt, 1999). Yen and Yen (2012) investigate the inside drivers motivating organizations to utilize green activities such as the top management commitment and relationships with vendors. The authors found a direct association between the proposed constructs of the study.

Furthermore, Gholami et al. (2013) examined senior managers’ perceptions about situations and the significances of using green practices. They presented that green technology acceptance, top management attitude, and apprehension for potential concerns are significantly interrelated. Moreover, they found an optimistic connection between the adoption of green practices and overall performance. The results from Cao and Chen (2018) study show that when the top management’s awareness increases, the association between coercive policies and GI strategy becomes stronger. Soewarno et al. (2019) propose that executives are responsible for making GI strategies that have to be implemented by employees. Such innovation strategies positively influence GI if applied appropriately. Thus, we propose hypothesis 3:

H_3_: EC has a significant impact on GI practices.

#### Environmental Performance

In this study, we have assessed the firms’ overall performance into two types: environmental and organizational. Environmental performance (EP) can be defined as “the environmental impact of a company’s activities on the natural surroundings” (Klassen and Whybark, 1999). OP includes numerous elements, both financial and non-financial (e.g., market share, reputation, sales volume, stakeholders satisfaction, etc.) (Venkatraman and Ramanujam, 1986).

Environmental performance encompasses the inclusion of eco-friendly ingredients in products, less pollution, reduced carbon emissions and waste at the source, advancements in energy-savings, efficiency in utilization of resources, reduction in the use of environmentally hazardous elements, etc. (Zhu et al., 2010). Related to long-term ecological impacts, an organization’s regulatory methods, processes, practices including pollution protection, as well as resource utilization and waste lessening, are more fruitful than “end-of-pipeline solutions” (Sarkis and Cordeiro, 2001; De Giovanni, 2012; Khan et al., 2019). Previous scholarships proposed that advancement in the production process and efficiency will upsurge opportunities to advance environmental performance (Montabon et al., 2007). Along with these, a study by Seman et al. (2019) on the 123-manufacturing industry showed that GI practices significantly improve environmental performance. Hence, we established hypothesis 4:

H_4_: GI practices have a significant impact on environmental performance.

#### Organizational Performance

Organizational performance can be assessed both “financially and non-financially” (Gounaris et al., 2003). To control environmental costs, organizations raise their productivity by adopting GI practices (de Burgos-Jiménez et al., 2013). Similarly, organizations can establish new markets and upsurge their market share by employing and adopting environmental activities and practices (Berry and Rondinelli, 1998; Berrone et al., 2017). A long-term organization goal, advancement into non-monetary performance can be demonstrated by enlarged customer loyalty, newly joined customers, and an improved image and reputation of an organization (Blazevic and Lievens, 2004). Chen (2008a) suggested that innovators in GI will gain the “first-mover advantage,” which indicates an improved firm image, higher product prices, competitive advantages, and new market opportunities. A study by Tang et al. (2018) shows that GI practices have positive effects on OP. Moreover, a study by Zhang and Walton (2017) on 83 New Zealand firms concludes that GI has a positive influence on the firms’ performance. Thus, hypothesis 5 is constructed:

Hypothesis 5: GI practices have a significant impact on OP.

This study used IO as a moderator. It tested its effect on the association among EC and GI practices because the variable is allied with organizations’ policy settings and culture, which primarily correlate to the firm’s employees.

#### Innovation Orientation

Innovation orientation is a strategic orientation that disturbs firms’ innovation practices and functions as a guiding standard for making strategy and enactment to increase an organization’s innovativeness (Chen et al., 2011; Stock and Zacharias, 2011). It defines a firms’ “openness to new ideas, technologies, skills, resources, and administrative systems” (Zhou et al., 2005) and a knowledge-sharing system that unites a learning viewpoint, strategic guidelines, and *trans*-functional acclimation within a firm to encourage innovation (Siguaw et al., 2006). IO is a crucial factor in overwhelming competitors and advancing an organization’s capability to effectively execute new products, services, systems, and processes (Oke, 2007). Organizations with a new innovative environment and management will motivate and encourage employees to commence innovative conduct (Ramus, 2018). Thus, we assume that an IO can advance the association between EC and GI practices, as exemplified in hypothesis 6:

H_6_: IO significantly moderates EC on GI practices.

## Research Methodology

### Instrument

Based on a review of the literature, we considered a structured closed-ended questionnaire with 7 s. The first section includes the demographical information of respondents. The second to seventh sections include the measurement items related to specific construct’s competitors’ pressure, governmental pressure; EC; IO; GI practices; environmental performance, and OP. To ensure the validity of the questionnaire and data, two pilot studies were conducted. After that step, we adopted a field survey on a large scale. All of the construct’s items were measured using “five-point Likert-type scales in which 1 = strongly disagree, 5 = strongly agree.”

### Data Collection and Sample

Data were collected from January 2019 to July 2019 from the manufacturing and services firms of Punjab province in Pakistan that have adopted GI practices. Convenient random sampling techniques were adopted for selecting areas of the country. Most of the organizations are based in Lahore, Faisalabad, Sheikhupura, Gujranwala, and Multan. Data collected by field surveys targeted the population, including the executives of different departments such as marketing, human resource, productions, operations, and other functional managers. After the pilot study’s conduction, 550 questionnaires were distributed among the respondents, out of which 520 were filled and returned. This resulted in a response rate of 94.54% from a random sampling method for data collection. Five forms were removed from the analysis due to incomplete information, and the remaining 515 were used in the analysis.

### Measures of the Constructs

This study adopted a quantitative research technique and a closed-ended questionnaire used for data collection. All of the variables were assessed with multiple-item scales. In total, 46 question items, mainly related to the constructs, were used. Competitor pressure was appraised by acclimating four items from preceding studies (Christmann, 2004). GP were measured by four items scale adapted from the studies of Zeng et al. (2011) and Qi et al. (2010). EC was measured by four items scale taken from Lindell and Karagozoglu (2001) studies and López-Gamero et al. (2008). IO was measured by seven items scale gained from the studies of Hurley and Hult (1998); Zhou et al. (2005), and Siguaw et al. (2006). In this study, GI practices were measured by nine items scale taken from the study of Chiou et al. (2011). OP measured by eight items scale adapted from the study of Blazevic and Lievens (2004) and Avlonitis et al. (2001). Moreover, the environmental performance was measured by six items scale adapted from Lin (2013) studies.

### Common Method Bias

We used Harman’s single factor test to check the issue of common method bias in the data. As per Harman’s methodology, if all the factors merged into factor analysis, and the first factor explains more than 50% of the data variance, there is an issue of common method bias. Therefore, we used the dimension reduction method in SPSS and merged all the factors into one factor using a rotation matrix. The first factor’s results explained 38.23% of the total variance, which is less than 50% of the variance. Thus, common method bias is not considered as the problem in this study.

## Data Analysis and Results

This study used the partial least squares (PLS) procedure of structural equation modeling using Smart-PLS Version 3.0 to assess the research model. This procedure was designated due to the investigative nature of the study (Hair et al., 2011). As recommended by Hair et al. (2013), this research applied a two-step method for statistical analysis. In the first step, the measurement model was analyzed. In the second step, the structural relationships among the latent constructs were assessed. This tactic was used to conclude both the reliability and validity of the theoretical variables before the model’s structural relationship was tested. Furthermore, Smart-PLS’s main reason includes the extensive popularity and acceptability of its application (Hair et al., 2012). It also includes comprehensive information about the variables (Hair et al., 2011).

### Sample Demographics

A sample of 515 employees represents the telecommunication sector population in China, and demographical representation was shown in Table 1. 392 (76.1%) respondents are male, and the rest, 123 (23.9%) respondents are female. Also, 246 (47.8%) respondents fall in the range of 31–40 years, followed by 219 (42.5%) in 20–30 years. From the education perspective, 291 (56.5%) respondents have a master’s degree, followed by 216 (41.9%) with a graduation degree, and the remaining (1.6%) with higher than master degree education, respectively. Furthermore, 218 (42.3%) respondents have a job in the sales and marketing department, 209 (40.6%) selected “other options,” apart from the HR and finance department. As for work experience, 260 (50.5%) respondents have 5–10 years of experience, followed by 127 (24.7%) with 1–5 years and the rest (24.3%) with 11–15 years of experience, respectively. As mentioned in the table below, 168 (32.6%) respondents have a monthly income of more than 60,000 rupees. Out of 515 respondents, 333 (64.7%) are married, and the rest, 182 (35.3%), are single.

**TABLE 1:** Demographical information.

	**Frequency**	**%**
**Gender**		
Male	392	**76.1**
Female	123	23.9
Total	515	100
**Age**		
20–30	219	42.5
31–40	246	**47.8**
41–50	50	9.7
Total	515	100
**Education**		
Graduation	216	41.9
*Master’s Degree*	291	**56.5**
Higher Than Master’s Degree	8	1.6
Total	515	100
**Department**		
HR	35	6.8
Financial	8	1.6
*Sales and Marketing*	218	**42.3**
Other	209	40.6
Total	515	100
**Work Experience**		
1–5 years	127	24.7
*5*–*10 Years*	260	**50.5**
11–15 Years	125	24.3
Total	515	100
**Salary (Rupees)**		
Below 20,000	11	2.1
40,000–60,000	159	30.9
*Above 60,000*	168	**32.6**
Total	515	100
**Marital Status**		
*Married*	333	**64.7**
Single	182	35.3
Total	515	100

*Bold values are the highest percentage values.*

### Measurement of Model

The partial least square method was used to measure the reliability and validity of the respective constructs. The constructs’ internal reliability was evaluated by “Cronbach’s Alpha (CA), and Composite reliability.” According to Gefen et al. (2000) and Hair et al. (2013), CA should be greater than 0.7. Moreover, Hinton (2014) categorized four ranges of CA. First, if the value falls in the range of 0.9, it falls in the area of excellent reliability. Second, if it falls between 0.7 and 0.9, it will have high reliability. Third, if it is in the range of 0.5 to 0.7, it will fall into the moderate area. Fourth, if it is <0.5, it will be categorized as low. Table 2 shows that all of the variables have values (e.g., CP = 0.851; GP = 0.829; EC = 0.851; IO = 0.764; GIP = 0.829; EP = 0.799; and OP = 0.892) which fall into the range of high reliability. Furthermore, to evaluate the convergent validity, the average variance extracted (AVE) is used. Fornell and Larcker (1981) and Bagozzi and Yi (1988) propose that AVE’s value should be greater than 0.5. As per results found in the table, all the values of constructs (0.691; 0.654; 0.627; 0.585; 0.598; 0.651; and 0.650) satisfied the rule of thumb. Chin (1998) recommended that loadings have a value greater than 0.5 because it indicates the constructs’ reliability. The item’s value can be between 0.4 and 0.7, as the value is also used by Umrani et al. (2018). Hence, all the loading values are found in the range of 0.477 to 0.894. Hence, it is proved that all the values satisfied the rule of thumb established by the scholars.

**TABLE 2:** Measurement model.

**Constructs**	**Items**	**Loadings**	**CA**	**CR**	**AVE**
Competitor Pressure			0.851	0.899	0.691
Industry initiatives/associations advocate the simple mentation of worldwide environmental standards by firms.	CP1	0.810			
Our major competitors set worldwide environmental standards for their operations and products.	CP2	0.829			
Our major competitors implement environmental strategies on a worldwide basis.	CP3	0.857			
Environmental strategies that we implement in one country affect considerably our environmental reputation with competitors in other countries.	CP4	0.828			
Government Pressure			0.829	0.882	0.654
Regulation for green construction is stringent.	GP1	0.894			
Future regulation for green construction is predictable.	GP2	0.699			
Regulations for green constructions have considerable impact on business entities.	GP3	0.798			
Regulations for green constructions effectively deal with issue regarding greening of construction process.	GP4	0.832			
Employees Conduct			0.851	0.894	0.627
The top management’s behavior inspired the acceptance of change by all the other organization members.	EP1	0.810			
The employees were able to take initiatives and decisions on their own thanks to the encouragement of authority delegation.	EP2	0.833			
The employees were aware of the progress made in their work are as new knowledge, new practice development.	EP3	0.803			
All the organization members knew and shared the firm’s mission and objectives.	EP4	0.746			
Innovation orientation			0.764	0.848	0.585
Technical innovation, based on research results, is readily accepted.	IO1	0.737			
Management actively seeks innovative ideas.	IO2	0.660			
Innovation is readily accepted in program/project management.	IO3	0.823			
People are penalized for new ideas that don’t work.	IO4	0.826			
Our firm pays close attention to innovation.	IO5	0.762			
Our firm emphasizes the need for innovation for development.	IO6	0.742			
Our firm promotes the need for development and utilization of new resources.	IO7	0.822			
Green Innovation Practices					
Lower consumption of e.g., water, electricity, gas, and petrol during production/use/disposal.	GIP1	0.809	0.829	0.881	0.598
Recycle, reuse, and remanufacture materials or parts.	GIP2	0.863			
Use of cleaner or renewable technology to make savings (such as energy, water, waste.)	GIP3	0.698			
Redesign of production and operation processes to improve environmental efficiency.	GIP4	0.666			
Redesigning and improving products or services to meet new environmental criteria or directives.	GIP5	0.813			
The company uses less or non-polluting/toxic materials that are environmentally friendly.	GIP6	0.852			
The Company uses materials that are easy to recycle, reuses, and decompose.	GIP7	0.782			
The Company recovers company’s end-of-life products and recycling.	GIP8	0.721			
The company uses eco-labeling.	GIP9	0.790			
Organizational Performance			0.892	0.918	0.650
The use of green innovation increased your sales directly (form environmental friendly products).	OP1	0.800			
The use of green product increased your overall sales (from other types of products as well).	OP2	0.846			
The use of green innovation preserved your current customers.	OP3	0.826			
The use of green innovation attracted new customers.	OP4	0.737			
The use of green innovation increased your market share.	OP5	0.832			
The use of green innovation increased your overall profitability.	OP6	0.791			
The use of green innovation enhanced the financial position of the firm.	OP7	0.812			
The use of green innovation enhanced the firm’s mental image among customers.	OP8	0.784			
Environmental Performance			0.799	0.877	0.651
Reduction of air emission.	EP1	0.890			
Reduction of hazardous waste/scrap.	EP2	0.889			
Reduction in consumption of gasoline/fuel.	EP3	0.891			
Partnership with green organizations and suppliers.	EP4	0.852			
Improvement of environmental compliance.	EP5	0.799			
Use of environmentally friendly material.	EP6	0.762			

*CA, Cronbach’s Alpha; CR, Composite Reliability; AVE, Average Variance Extracted.*

Two methods were used to evaluate the discriminant validity (e.g., used to measure either construct used in the study well defined). Each construct is pure and not any multicollinearity involved. The dependent variable was evaluated by considering the correlations between the measures of hypothetically intersecting variables) of the variables. First, it was ensured that the cross-loadings of indicators should be greater than any other opposing constructs (Hair et al., 2012). Second, according to the criterion of Anderson and Gerbing (1988) and Fornell and Larcker (1981), the “square root of AVE for each construct should exceed the inter-correlations of the construct with other model constructs” (Table 3). Hence, both methods ensured the satisfaction of the results and validity. All the results found in the study meet satisfactory status.

**TABLE 3:** Discriminant validity coefficients.

	**1**	**2**	**3**	**4**	**5**	**6**	**7**
CP	0.831*						
EC	0.751	0.792*					
EP	0.606	0.462	0.807*				
GP	0.50	0.493	0.42	0.809*			
GIP	0.777	0.705	0.563	0.544	0.773*		
IO	0.802	0.684	0.517	0.478	0.709	0.765*	
OP	0.465	0.502	0.429	0.797	0.562	0.472	0.806*

*Bold values represent the square root of average variance extracted (AVE).

Another essential technique of partial least square to assess the model’s validity and multicollinearity includes the Heterotrait–Monotrait ratio. According to Henseler et al. (2015). HTMT is the ratio of trait correlation to within correlation. The belief that if the HTMT value is going to increase >0.9, it will lack the discriminant validity, as mentioned in Table 4. Furthermore, it is considered one of the most crucial technique to measure the multicollinearity.

**TABLE 4:** Heterotrait – Monotrait (HTMT) ratio.

	**1**	**2**	**3**	**4**	**5**	**6**	**7**
Competitor pressure							
Employee conduct	0.846						
Environmental performance	0.604	0.493					
Governmental pressure	0.444	0.478	0.351				
Green Innovation Practices	0.778	0.717	0.55	0.486			
Innovation orientation	0.667	0.65	0.424	0.379	0.75		
Organizational performance	0.496	0.572	0.454	0.749	0.614	0.445	

### Structural Model

The table given below contains the values of the coefficient of determination. It shows the percentage change in the dependent variable incurred because of independent variables. Hair et al. (2010) defined it as the proportion determined by independent variables. In other words, it tells how much change in dependent variable incurs because of the independent variable. Table 5 shows three models. In the path – 1: R^2^ of GI practice, have a positive coefficient 0.716, and adjusted R^2^ 0.713. It entails that 71.6% of changes in GIP incur because of all the independent variables. Path – 2 exhibited a 31.7% change in EP. While path – 3 showed a 31.6% change in OP incurred because of all the independent variables. According to Hair et al. (2011) and Henseler et al. (2015), three values of the coefficient of determination, 0.75, 0.5, or 0.25, which are called substantial, moderate, or weak, respectively. If the co-efficient of determination falls within the range of 0.75 or greater, it will become significant. If it is between 0.25 and 0.75, it will become moderate. If it falls below 0.25, it will be considered weak. Hence, the study’s value, which is shown in the table underneath, falls in a moderate range.

**TABLE 5:** Analysis of R^2^.

**Path**	**R square**	**R square adjusted**	**Decision**
1. GI practices	0.716	0.713	Moderate
2. Environmental performance	0.317	0.315	Moderate
3. Organizational performance	0.316	0.315	Moderate

### Analysis and Discussion

The competitors’ pressure, governmental pressure, EC, and GI practices are concentrated on environmental and OP. The manufacturing and servicing industries of the country were examined, which account for greater than 70% contribution to the GDP of the country. A cohesive framework was developed under the investigation of theory, and it stated that the stakeholders’ dimensions have positive and significant effects on the GI practice, and which, in turn, has positive and significant impacts on environmental and OP.

**FIGURE 2:**
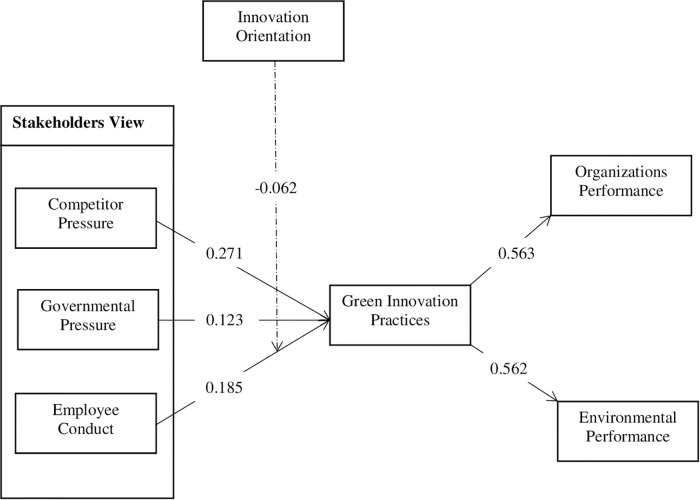
Structural model of the study.

In the study, six hypotheses were constructed. Among them, five were a direct hypothesis, and one was proposed for the moderation effect. As exhibited in Table 6 and Figure 2, the first direct hypothesis H_1_ related to the influence of competitor pressure on GI practices. The findings show that competitive pressure positively and significantly impacts GI practices with a coefficient value of 0.271, *t*-value 5.543 > 2, and *p*-value 0.000 < 0.05. The hypothesis results were found consistent with the study of El-Kassar and Singh (2019). Moreover, we tested H_2_ governmental pressure positively related to GI practices. The results indicate that governmental pressure positively and significantly impacts GI practices with a positive coefficient value of 0.123, *t*-value 4.598 > 2, and *p*-value 0.000 < 0.05. The second direct hypothesis H_2_, won the vote of support and was consistent with the results from a previous study of Sezen and Çankaya (2013) and Fernando and Wah (2017). Our third hypothesis, H3, is associated with EC and GI practices. The output illustrates that EC positively influenced GI practices with coefficient value of 0.185, *t*-value 4.368 > 2, and *p*-value 0.000 < 0.05. Hypothesis results were found consistent with the study of Yen and Yen (2012), Gholami et al. (2013), and Soewarno et al. (2019).

**TABLE 6:** Path coefficients and hypothesis testing.

**Hypothesis**	**Relationship**	**Path coefficient**	**S. D**	***t*-value**	***p*-value**	**Decision**
***Direct effect***						
H_1_	CP→GIP	0.271	0.049	5.543	0.000**	Supported
H_2_	GP→GIP	0.123	0.027	4.598	0.000**	Supported
H_3_	EC→GIP	0.185	0.042	4.368	0.000**	Supported
H_4_	GIP→EP	0.563	0.038	14.653	0.000**	Supported
H_5_	GIP→OP	0.562	0.035	16.15	0.000**	Supported
***Moderating effect***						
H_6_	IO × EC→GIP	–0.063	0.02	3.137	0.002	Supported

**p-value = 0.05, *t*-value = 2.

**FIGURE 3:**
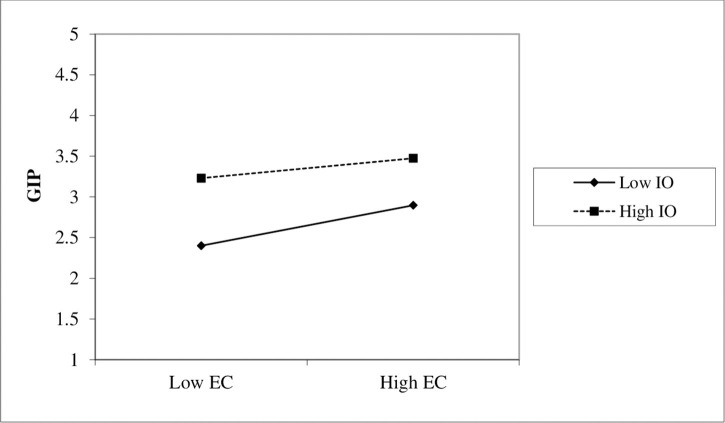
Interaction graph EC × IO and GIP.

Furthermore, we discussed the H_4_ the direct effect of GI practices on OP. The findings show that GI practices positively and significantly affect OP with a positive coefficient value of 0.563, *t*-value 14.653 > 2, and *p*-value 0.000 < 0.05. Hypothesis results were consistent with the previous study of Seman et al. (2019). Besides, we tested the direct effect of GI practices on environmental performance. We found that GI practices positively related to environmental performance with a positive coefficient of 0.562, *t*-value 16.15 > 2, and *p*-value 0.000 < 0.05. The hypothesis was supported and consistent with the studies of Zhang and Walton (2017) and Tang et al. (2018). Finally, the sixth hypothesis H_6_ was constructed for moderation interaction effects, and its results were found statistically significant with a negative coefficient value of −0.063, *t*-value 3.137 > 2, and *p*-value 0.000 < 0.05. In conclusion, the results of all direct hypotheses were found with a positive path coefficient and statistically significant with a *t*-value > 2 and *p*-value < 0.05 and the interaction graph presented in Figure 3. However, the moderation hypothesis was found statistically significant, with a negative coefficient value. Therefore, it is proven that all the variables used in the study affect GI practices and the firms’ overall performance.

## Conclusion and Implications

### Conclusion

“Go green” has been forcing internationally dynamic organizations to improve their green competencies endlessly, execute GI practices to prevent the environment from degrading further, and advance overall firms’ performance. Therefore, this study aims to identify the key factors affecting on the GI practices and its impact on OP from stakeholders’ perspectives. From the results, it is concluded that competitive pressure has a positive and significant impact on GI practices (Abrahamson and Rosenkopf, 1993; Cai and Li, 2018; Durand and Georgallis, 2018; Singh and El-Kassar, 2018; Yu, 2019) as well as that governmental pressure has a positive and significant impact on GI practices (Lindell and Karagozoglu, 2001; Bernauer et al., 2007; Zeng et al., 2011; Huang et al., 2016; Fernando and Wah, 2017; Yakubu, 2017; Famiyeh et al., 2018; He et al., 2018; Tirabeni et al., 2019; Zhang et al., 2019). Furthermore, it can be seen from our results that employee’s conduct is positively influenced by GI practices (Reinhardt, 1999; Daily and Huang, 2001; Zhu et al., 2008; Yen and Yen, 2012; Gholami et al., 2013; Cao and Chen, 2018; Tang et al., 2018; Soewarno et al., 2019). Also, our results conclude that GI practices have a positive and significant effect on OP (Berry and Rondinelli, 1998; Gounaris et al., 2003; Blazevic and Lievens, 2004; Chen, 2008a; de Burgos-Jiménez et al., 2013; Berrone et al., 2017; Zhang and Walton, 2017; Tang et al., 2018). The findings of the study suggest that GI practices positively related to environmental performance. From the findings, it is also concluded that the moderation effect of IO was found statistically significant but with a negative coefficient value. The study also describes significant implications and suggestions to the managers and policymakers.

### Implications

The present study delivers numerous researches “contributions and managerial implications.” First, this study presented that GI practices disturb not only EP but also OP. GI should be seen not only as responsive contentment of management requirements but as a pre-emptive exercise to advance a competitive advantage and the firm’s performance (de Burgos-Jiménez et al., 2013). This pragmatic sign proposes that when organizations generously emphasize GI practices, they can promote both “financial and non-financial” performance. Top management executives can play a crucial role in carrying the significance of GI to all stakeholders. Second, both industrial and service organizations were investigated in the model. The data collected from both the sectors/industries showed no difference, and the results were the same. “Go green” is a significant issue for both divisions. GI practices need to be endlessly accepted in the product, process, marketing, management innovation, or all, regardless of industry. Finally, this study showed a statistically significant moderation effect of IO on EC concerning GI practices. However, we propose that the top management or executives accentuate innovation and inventiveness in their firm’s culture. The effort to raise the constituents of innovation is critical to the existence and sustainability of firms.

## Limitations and Further Research

Although this research study delivers valuable intuitions, some limitations should fuel further investigations. First, the study was conducted in Pakistan, which only included significant areas of the country; small cities were ignored in the research. Second, an executive’s insights into GI practices and consequences are stranded in specific-industry norms. However, to focus on the conclusions’ larger generalizability, we invite scholars to replicate our study but in diverse perspectives and countries. Future studies should include other dimensions of the stakeholders’ view with the mediation of market innovation and management innovation. HR practices can also moderate the relationship between stakeholders’ views and GI practices. Last, the mediation effects need to be explored further.

## Data Availability Statement

The raw data supporting the conclusions of this article will be made available by the authors, without undue reservation.

## Ethics Statement

This study was carried out in accordance with the recommendations of the Ethical Principles of Psychologists and Code of Conduct of the American Psychological Association (APA). All participants gave written consent in accordance with the Declaration of Helsinki. The studies involving human participants were reviewed and approved by the Ethics Committee of the Lahore School of Business, University of Lahore, Pakistan. The patients/participants provided their written informed consent to participate in this study.

## Author Contributions

MK, HW, and DA: the provision of materials (i.e., questionnaires) and principal manuscript writing. MM, FS, and FA: data collection and manuscript revision and proofreading. MK and HW: data analysis plan. FS and FA: data analysis. All authors contributed to definition of research objectives, models, and hypotheses and approved the final version of the manuscript.

## Conflict of Interest

The authors declare that the research was conducted in the absence of any commercial or financial relationships that could be construed as a potential conflict of interest.

## Annexure

Descriptive statistics.

**TABLE:**

**Items**	**Mean**	**Median**	**Min**	**Max**	**SD**	**Excess Kurtosis**	**Skewness**
OP1	3.737	4	1	5	0.799	0.195	–0.319
OP2	4.171	4	2	5	0.835	–0.192	–0.731
OP3	4.029	4	2	5	0.837	–0.456	–0.494
OP4	4.05	4	2	5	0.794	–0.629	–0.371
OP5	3.775	4	1	5	0.914	0.086	–0.581
OP6	3.99	4	1	5	0.906	–0.45	–0.499
OP7	4.031	4	1	5	0.883	–0.131	–0.621
OP8	2.324	3	1	5	0.722	–0.192	–0.571
GP1	4.072	4	1	5	0.935	–0.158	–0.729
GP2	3.897	4	1	5	1.01	0.207	–0.756
GP3	3.763	4	1	5	0.998	–1.054	–0.193
GP4	4.004	4	1	5	0.929	–0.666	–0.519
EP1	3.802	4	1	5	1.033	–0.005	–0.687
EP2	3.981	4	1	5	0.997	0.239	–0.869
EP3	4.023	4	1	5	0.918	0.721	–0.863
EP4	3.353	3	1	5	1.139	–0.4	–0.407
EP5	3.821	4	1	5	0.821	–0.213	–0.731
EP6	4.721	4	1	5	0.945	–0.172	–0.261
CP1	3.82	4	1	5	0.864	0.582	–0.624
CP2	3.946	4	1	5	0.893	–0.184	–0.484
CP3	3.697	4	1	5	0.9	–0.304	–0.441
CP4	4.014	4	1	5	0.936	0.298	–0.783
IO1	3.928	4	1	5	0.986	1.148	–1.064
IO2	2.92	3	1	5	1.339	–1.155	0.01
IO3	2.792	3	1	5	1.311	–1.253	–0.001
IO4	3.779	4	1	5	1.117	–0.232	–0.745
IO5	3.975	4	1	5	0.962	–0.404	–0.632
IO6	4.002	4	1	5	1.032	0.339	–0.938
IO7	3.272	3	1	5	1.134	–0.467	–0.508
EC1	3.831	4	2	5	0.771	–0.518	–0.133
EC2	3.817	4	2	5	0.736	–0.042	–0.314
EC3	3.802	4	1	5	0.868	0.322	–0.535
EC4	3.852	4	2	5	0.869	–0.687	–0.28
GIP1	3.579	4	1	5	1.037	0.453	–0.819
GIP2	3.371	4	1	5	1.267	–0.688	–0.579
GIP3	3.495	4	1	5	0.929	0.267	–0.597
GIP4	3.338	4	1	5	1.105	–0.335	–0.568
GIP5	3.621	4	1	5	0.915	0.767	–0.796
GIP6	4.068	4	2	5	0.884	–0.226	–0.708
GIP7	3.252	3	1	5	1.064	–0.507	–0.216
GIP8	3.724	4	1	5	0.869	0.944	–0.699
GIP9	3.864	4	1	5	0.871	0.459	–0.636

## References

[B1] Abrahamson E. Rosenkopf L. (1993). Institutional and competitive bandwagons: using mathematical modeling as a tool to explore innovation diffusion. *Acad. Manage. Rev.* 18 487–517. 10.5465/amr.1993.9309035148

[B2] Afridi S. A. Afsar B. Shahjehan A. Khan W. Rehman Z. U. Khan M. A. S. (2020). Impact of corporate social responsibility attributions on employee’s extra role behaviors: moderating role of ethical corporate identity and interpersonal trust. *Corpor. Soc. Responsib. Environ. Manage.* 2020 1–14.

[B3] Albort-Morant G. Leal-Millán A. Cepeda-Carrion G. Henseler J. (2018). Developing green innovation performance by fostering of organizational knowledge and coopetitive relations. *Rev. Manage. Sci.* 12 499–517. 10.1007/s11846-017-0270-z

[B4] Alrowwad A. A. Obeidat B. Y. Tarhini A. Aqqad N. (2017). The impact of transformational leadership on organizational performance via the mediating role of corporate social responsibility: a structural equation modeling approach. *Int. Bus. Res.* 10 199–221. 10.5539/ibr.v10n1p199

[B5] Anderson J. C. Gerbing D. W. (1988). Structural equation modeling in practice: a review and recommended two-step approach. *Psychol. Bulletin* 103:411 10.1037/0033-2909.103.3.411

[B6] Arfi W. B. Hikkerova L. Sahut J.-M. (2018). External knowledge sources, green innovation and performance. *Technol. Forecasting Soc. Change* 129 210–220. 10.1016/j.techfore.2017.09.017

[B7] Atlin C. Gibson R. (2017). Lasting regional gains from non-renewable resource extraction: the role of sustainability-based cumulative effects assessment and regional planning for mining development in canada. *Extr. Ind. Soc.* 4 36–52. 10.1016/j.exis.2017.01.005

[B8] Avlonitis G. J. Papastathopoulou P. G. Gounaris S. P. (2001). An empirically-based typology of product innovativeness for new financial services: Success and failure scenarios. *J. Prod. Innovation Manage.* 18 324–342. 10.1111/1540-5885.1850324

[B9] Bagozzi R. P. Yi Y. (1988). On the evaluation of structural equation models. *J. Acad. Marketing Sci.* 16 74–94.

[B10] Bernauer T. Engel S. Kammerer D. Sejas Nogareda J. (2007). Explaining green innovation: ten years after Porter’s win-win proposition: how to study the effects of regulation on corporate environmental innovation? *Politisc. Vierteljahresschrift* 39 323–341.

[B11] Berrone P. Fosfuri A. Gelabert L. (2017). Does greenwashing pay off? Understanding the relationship between environmental actions and environmental legitimacy. *J. Bus. Ethics* 144 363–379. 10.1007/s10551-015-2816-9

[B12] Berry M. A. Rondinelli D. A. (1998). Proactive corporate environmental management: A new industrial revolution. *Acad. Manage. Perspect.* 12 38–50. 10.5465/ame.1998.650515

[B13] Blazevic V. Lievens A. (2004). Learning during the new financial service innovation process: antecedents and performance effects. *J. Bus. Res.* 57 374–391. 10.1016/s0148-2963(02)00272-2

[B14] Bryson J. M. (2018). *Strategic planning for public and nonprofit organizations: A guide to strengthening and sustaining organizational achievement.* Hoboken, NJ: John Wiley & Sons.

[B15] Buysse K. Verbeke A. (2003). Proactive environmental strategies: a stakeholder management perspective. *Strategic Manage. J.* 24 453–470. 10.1002/smj.299

[B16] Cai W. Li G. (2018). The drivers of eco-innovation and its impact on performance: evidence from China. *J. Cleaner Prod.* 176 110–118. 10.1016/j.jclepro.2017.12.109

[B17] Cao H. Chen Z. (2018). The driving effect of internal and external environment on green innovation strategy-The moderating role of top management’s environmental awareness. *Nankai Bus. Rev. Int.* 10 342–361. 10.1108/nbri-05-2018-0028

[B18] Chan H. K. He H. Wang W. Y. (2012). Green marketing and its impact on supply chain management in industrial markets. *Ind. Marketing Manage.* 41 557–562. 10.1016/j.indmarman.2012.04.002

[B19] Chan H.-L. Shen B. Cai Y. (2018). Quick response strategy with cleaner technology in a supply chain: coordination and win-win situation analysis. *Int. J. Prod. Res.* 56 3397–3408. 10.1080/00207543.2016.1278283

[B20] Chang C.-H. (2011). The influence of corporate environmental ethics on competitive advantage: the mediation role of green innovation. *J. Bus. Ethics* 104 361–370. 10.1007/s10551-011-0914-x

[B21] Chen J.-S. Tsou H.-T. Ching R. K. (2011). Co-production and its effects on service innovation. *Ind. Marketing Manage.* 40 1331–1346. 10.1016/j.indmarman.2011.03.001

[B22] Chen Y.-S. (2008a). The driver of green innovation and green image–green core competence. *J. Bus. Ethics* 81 531–543. 10.1007/s10551-007-9522-1

[B23] Chen Y.-S. (2008b). The positive effect of green intellectual capital on competitive advantages of firms. *J. Bus. Ethics* 77 271–286. 10.1007/s10551-006-9349-1

[B24] Chen Y.-S. Lai S.-B. Wen C.-T. (2006). The influence of green innovation performance on corporate advantage in Taiwan. *J. Bus. Ethics* 67 331–339. 10.1007/s10551-006-9025-5

[B25] Chin W. W. (1998). The partial least squares approach to structural equation modeling. *Mod. Methods Bus. Res.* 295 295–336.

[B26] Chiou T.-Y. Chan H. K. Lettice F. Chung S. H. (2011). The influence of greening the suppliers and green innovation on environmental performance and competitive advantage in taiwan. *Transp. Res. Part E Logist. Transp. Rev.* 47 822–836. 10.1016/j.tre.2011.05.016

[B27] Christmann P. (2004). Multinational companies and the natural environment: determinants of global environmental policy. *Acad. Manage. J.* 47 747–760. 10.5465/20159616

[B28] Chu Z. Wang L. Lai F. (2019). Customer pressure and green innovations at third party logistics providers in china. *Int. J. Logist. Manage.* 30 57–75. 10.1108/ijlm-11-2017-0294

[B29] Clarkson M. E. (1995). A stakeholder framework for analyzing and evaluating corporate social performance. *Acad. Manage. Rev.* 20 92–117. 10.5465/amr.1995.9503271994

[B30] Claver E. Lopez M. D. Molina J. F. Tarí J. J. (2007). Environmental management and firm performance: a case study. *J. Environ. Manage.* 84 606–619. 10.1016/j.jenvman.2006.09.012 17141938

[B31] Co H. Barro F. (2009). Stakeholder theory and dynamics in supply chain collaboration. *Int. J. Oper. Prod. Manage.* 29 591–611. 10.1108/01443570910957573

[B32] Cordano M. Marshall R. S. Silverman M. (2010). How do small and medium enterprises go “green”? A study of environmental management programs in the US wine industry. *J. Bus. Ethics* 92 463–478. 10.1007/s10551-009-0168-z

[B33] Crane A. Livesey S. (2017). “Are you talking to me?: stakeholder communication and the risks and rewards of dialogue,” in *Unfolding stakeholder thinking 2*, eds Andriof J. Waddock S. Husted B. Rahman S. S. (New York, NY: Routledge), 39–52. 10.9774/gleaf.978-1-909493-32-2_4

[B34] Daily B. F. Huang S.-C. (2001). Achieving sustainability through attention to human resource factors in environmental management. *Int. J. Oper. Prod. Manage.* 21 1539–1552. 10.1108/01443570110410892

[B35] de Burgos-Jiménez J. Vázquez-Brust D. Plaza-Úbeda J. A. Dijkshoorn J. (2013). Environmental protection and financial performance: an empirical analysis in wales. *Int. J. Oper. Prod. Manage.* 33 981–1018. 10.1108/ijopm-11-2010-0374

[B36] De Giovanni P. (2012). Do internal and external environmental management contribute to the triple bottom line? *Int. J. Oper. Prod. Manage.* 32 265–290. 10.1108/01443571211212574

[B37] Donaldson T. Preston L. E. (1995). The stakeholder theory of the corporation: concepts, evidence, and implications. *Acad. Manage. Rev.* 20 65–91. 10.2307/258887

[B38] Durand R. Georgallis P. (2018). Differential firm commitment to industries supported by social movement organizations. *Organ. Sci.* 29 154–171. 10.1287/orsc.2017.1170 19642375

[B39] El-Kassar A.-N. Singh S. K. (2019). Green innovation and organizational performance: the influence of big data and the moderating role of management commitment and HR practices. *Technol. Forecast. Social Change* 144 483–498. 10.1016/j.techfore.2017.12.016

[B40] Famiyeh S. Adaku E. Amoako-Gyampah K. Asante-Darko D. Amoatey C. T. (2018). Environmental management practices, operational competitiveness and environmental performance: empirical evidence from a developing country. *J. Manuf. Technol. Manage.* 29 588–607. 10.1108/jmtm-06-2017-0124

[B41] Feng L. Zhao W. Li H. Song Y. (2018). The effect of environmental orientation on green innovation: do political ties matter? *Sustainability* 10:4674 10.3390/su10124674

[B42] Feng Z. Chen W. (2018). Environmental regulation, green innovation, and industrial green development: an empirical analysis based on the spatial durbin model. *Sustainability* 10:223 10.3390/su10010223

[B43] Fernando Y. Jabbour C. J. C. Wah W.-X. (2019). Pursuing green growth in technology firms through the connections between environmental innovation and sustainable business performance: Does service capability matter? *Resour. Conserv. Recyl.* 141 8–20. 10.1016/j.resconrec.2018.09.031

[B44] Fernando Y. Wah W. X. (2017). The impact of eco-innovation drivers on environmental performance: empirical results from the green technology sector in malaysia. *Sustainable Prod. Consumption* 12 27–43. 10.1016/j.spc.2017.05.002

[B45] Foo M. Y. (2018). *Green purchasing capabilities and practices towards triple bottom line performance: Moderating effects of institutional pressure/. Foo Meow Yee.* Phd Thesis. Kuala Lumpur: University of Malaya.

[B46] Fornell C. Larcker D. F. (1981). Evaluating structural equation models with unobservable variables and measurement error. *J. Marketing Res.* 18 39–50. 10.2307/3151312

[B47] Freeman E. (1984). Strategic Management. A Stakeholder Approach. *Stakehold. Manag. Framew. Phil.* 2 267–299.

[B48] Freeman R. E. (2010). *Strategic management: A stakeholder approach.* Cambridge: Cambridge university press.

[B49] Friedman A. L. Miles S. (2006). *Stakeholders: Theory and practice.* Oxford: Oxford University Press.

[B50] Gao Y. Tsai S.-B. Xue X. Ren T. Du X. Chen Q. (2018). An empirical study on green innovation efficiency in the green institutional environment. *Sustainability* 10:724 10.3390/su10030724

[B51] Gefen D. Straub D. Boudreau M.-C. (2000). Structural equation modeling and regression: Guidelines for research practice. *Comm. Assoc. Info. Sys.* 4:7.

[B52] Gholami R. Sulaiman A. B. Ramayah T. Molla A. (2013). Senior managers’ perception on green information systems (IS) adoption and environmental performance: results from a field survey. *Inf. Manag.* 50 431–438. 10.1016/j.im.2013.01.004

[B53] Gluch P. Gustafsson M. Thuvander L. (2009). An absorptive capacity model for green innovation and performance in the construction industry. *Constr. Manage. Eco.* 27 451–464. 10.1080/01446190902896645

[B54] Gounaris S. P. Papastathopoulou P. G. Avlonitis G. J. (2003). Assessing the importance of the development activities for successful new services: does innovativeness matter? *Int. J. Bank Marketing* 21 266–279. 10.1108/02652320310488448

[B55] Hair J. F. Anderson R. E. Babin B. J. Black W. C. (2010). *Multivariate data analysis: A global perspective (Vol. 7). In.* Upper Saddle River, NJ: Pearson.

[B56] Hair J. F. Ringle C. M. Sarstedt M. (2011). PLS-SEM: Indeed a silver bullet. *J. Marketing Theory Prac.* 19 139–152. 10.2753/mtp1069-6679190202

[B57] Hair J. F. Ringle C. M. Sarstedt M. (2013). Partial least squares structural equation modeling: rigorous applications, better results and higher acceptance. *Long Range plan.* 46 1–12. 10.1016/j.lrp.2013.01.001

[B58] Hair J. F. Sarstedt M. Pieper T. M. Ringle C. M. (2012). The use of partial least squares structural equation modeling in strategic management research: a review of past practices and recommendations for future applications. *Long Range plan.* 45 320–340. 10.1016/j.lrp.2012.09.008

[B59] He Z.-X. Shen W.-X. Li Q.-B. Xu S.-C. Zhao B. Long R.-Y. (2018). Investigating external and internal pressures on corporate environmental behavior in papermaking enterprises of China. *J. Clean.Prod.* 172 1193–1211. 10.1016/j.jclepro.2017.10.115

[B60] Henseler J. Ringle C. M. Sarstedt M. (2015). A new criterion for assessing discriminant validity in variance-based structural equation modeling. *J. Acad. Market. Sci.* 43 115–135. 10.1007/s11747-014-0403-8

[B61] Hillestad T. Xie C. Haugland S. A. (2010). Innovative corporate social responsibility: the founder’s role in creating a trustworthy corporate brand through “green innovation”. *J. Prod. Brand Manage.* 19 440–451. 10.1108/10610421011085758

[B62] Hinton P. R. (2014). *Statistics explained.* Abingdon: Routledge.

[B63] Ho Y.-H. Lin C.-Y. Chiang S.-H. (2009). Organizational determinants of green innovation implementation in the logistics industry. *Int. J. Organ. Innov.* 2 3.

[B64] Huang X.-x Hu Z.-p. Liu C.-s. Yu D.-j. Yu L.-f. (2016). The relationships between regulatory and customer pressure, green organizational responses, and green innovation performance. *J. Cleaner Prod.* 112 3423–3433. 10.1016/j.jclepro.2015.10.106

[B65] Huang Y.-C. Ding H.-B. Kao M.-R. (2009). Salient stakeholder voices: family business and green innovation adoption. *J. Manage.Organ.* 15 309–326. 10.5172/jmo.2009.15.3.309

[B66] Hurley R. F. Hult G. T. M. (1998). Innovation, market orientation, and organizational learning: an integration and empirical examination. *J. Marketing* 62 42–54. 10.1177/002224299806200303

[B67] Ilvitskaya S. Prihodko V. (2018). Innovative technologies in the field of topography, land management, territorial planning, construction and architecture. *IOP Conf. Ser. Mater. Sci. Eng.* 365:022030 10.1088/1757-899x/365/2/022030

[B68] Jaaffar A. H. Amran A. A. (2017). The influence of leaders’ past environmental-related experiences and positive deviance behaviour in green management practices. *J. Pengurusan* 51 1–18.

[B69] Jones T. M. Wicks A. C. (1999). Convergent stakeholder theory. *Acad.Manage. Rev.* 24 206–221. 10.2307/259075

[B70] Kammerer D. (2009). The effects of customer benefit and regulation on environmental product innovation.: empirical evidence from appliance manufacturers in germany. *Ecol. Eco.* 68 2285–2295. 10.1016/j.ecolecon.2009.02.016

[B71] Khan M. A. S. Ali M. Usman M. Saleem S. Jianguo D. (2019). Interrelationships between ethical leadership, green psychological climate, and organizational environmental citizenship behavior: the moderating role of gender. *Front. Psychol.* 10:1977. 10.3389/fpsyg.2019.01977 31555173 PMC6722205

[B72] Khan M. A. S. Jianguo D. Saleem S. Boamah K. B. Javed U. Usman M. (2019). Rejuvenating the concept of work alienation through job demands-resources model and examining its relationship with emotional exhaustion and explorative and exploitative learning. *Psychol. Res Behav. Manage.* 12 931. 10.2147/prbm.s204193 31632165 PMC6789178

[B73] Khan S. A. R. Qianli D. (2017). Impact of green supply chain management practices on firms’ performance: an empirical study from the perspective of Pakistan. *Environ. Sci. Pollu. Res.* 24 16829–16844. 10.1007/s11356-017-9172-5 28573559

[B74] Klassen R. D. Whybark D. C. (1999). The impact of environmental technologies on manufacturing performance. *Acad. Manage. J.* 42 599–615. 10.5465/256982

[B75] Laplume A. O. Sonpar K. Litz R. A. (2008). Stakeholder theory: reviewing a theory that moves us. *J. Manage.* 34 1152–1189. 10.1177/0149206308324322

[B76] Lee J. W. Kim Y. M. Kim Y. E. (2018). Antecedents of adopting corporate environmental responsibility and green practices. *J. Bus. Ethics* 148 397–409. 10.1007/s10551-016-3024-y

[B77] Lin C.-Y. Ho Y.-H. (2008). An empirical study on logistics service providers’ intention to adopt green innovations. *J. Technol. Manage.Innov.* 3 17–26.

[B78] Lin C.-Y. Ho Y.-H. (2011). Determinants of green practice adoption for logistics companies in China. *J. Bus. Ethics* 98 67–83. 10.1007/s10551-010-0535-9

[B79] Lin R.-J. (2013). Moderating effects of total quality environmental management on environmental performance. *Afr. J. Bus. Manag.* 5 8088–8099. 10.5897/AJBM10.1399

[B80] Lindell M. Karagozoglu N. (2001). Corporate environmental behaviour–a comparison between nrdic and US firms. *Bus. Strategy Environ.* 10 38–52. 10.1002/1099-0836(200101/02)10:1<38::aid-bse269>3.0.co;2-c

[B81] López-Gamero M. D. Claver-Cortés E. Molina-Azorín J. F. (2008). Complementary resources and capabilities for an ethical and environmental management: a qual/quan study. *J. Bus. Ethics* 82 701–732. 10.1007/s10551-007-9587-x

[B82] Mitchell R. K. Agle B. R. Wood D. J. (1997). Toward a theory of stakeholder identification and salience: defining the principle of who and what really counts. *Acad. Manage. Rev.* 22 853–886. 10.2307/259247

[B83] Montabon F. Sroufe R. Narasimhan R. (2007). An examination of corporate reporting, environmental management practices and firm performance. *J. Oper. Manage.* 25 998–1014. 10.1016/j.jom.2006.10.003

[B84] Murillo-Luna J. L. Garcés-Ayerbe C. Rivera-Torres P. (2008). Why do patterns of environmental response differ? A stakeholders’ pressure approach. *Strategic Manage. J.* 29 1225–1240. 10.1002/smj.711

[B85] Oke A. (2007). Innovation types and innovation management practices in service companies. *Int. J. Oper. Prod. Manage.* 27 564–587. 10.1108/01443570710750268

[B86] Porter M. E. Kramer M. R. (2019). *Creating shared value. In Managing sustainable business.* New York, NY.: Springer, 323–346.

[B87] Qi G. Shen L. Zeng S. Jorge O. J. (2010). The drivers for contractors’ green innovation: an industry perspective. *J. Cleaner Prod.* 18 1358–1365. 10.1016/j.jclepro.2010.04.017

[B88] Ramus C. A. (2018). *Employee environmental innovation in firms: Organizational and managerial factors.* Abingdon: Routledge.

[B89] Reinhardt F. L. (1999). Bringing the environment down to earth. *Harvard Bus. Rev.* 77 149–149. 10539206

[B90] Rennings K. (2000). Redefining innovation—eco-innovation research and the contribution from ecological economics. *Ecol. Eco.* 32 319–332. 10.1016/s0921-8009(99)00112-3

[B91] Rusinko C. (2007). Green manufacturing: an evaluation of environmentally sustainable manufacturing practices and their impact on competitive outcomes. *IEEE Trans. Eng. Manage.* 54 445–454. 10.1109/tem.2007.900806

[B92] Saeed A. Jun Y. Nubuor S. Priyankara H. Jayasuriya M. (2018). Institutional pressures, green supply chain management practices on environmental and economic performance: a two theory view. *Sustainability* 10 1517 10.3390/su10051517

[B93] Salem M. A. Shawtari F. Shamsudin M. F. Hussain H. B. I. (2018). The consequences of integrating stakeholder engagement in sustainable development (environmental perspectives). *Sustainable Dev.* 26 255–268. 10.1002/sd.1699

[B94] Sarkis J. Cordeiro J. J. (2001). An empirical evaluation of environmental efficiencies and firm performance: pollution prevention versus end-of-pipe practice. *European J. Oper. Res.* 135 102–113. 10.1016/s0377-2217(00)00306-4

[B95] Seman N. A. A. Govindan K. Mardani A. Zakuan N. Saman M. Z. M. Hooker R. E. (2019). The mediating effect of green innovation on the relationship between green supply chain management and environmental performance. *J. Cleaner Prod.* 229 115–127. 10.1016/j.jclepro.2019.03.211

[B96] Seman N. A. A. Zakuan N. Rashid U. K. Nasuredin J. Ahmad N. (2018). Understanding Stakeholder Pressures in Adopting Environmental Management Practices Based on Stakeholder Theory: A Review. *Int. J. Res.* 05 1530–1545.

[B97] Sezen B. Çankaya S. Y. (2013). Effects of green manufacturing and eco-innovation on sustainability performance. *Procedia Soc. Behav. Sci.* 99 154–163. 10.1016/j.sbspro.2013.10.481

[B98] Shahzad F. Du J. Khan I. Shahbaz M. Murad M. Khan M. S. S. (2020). Untangling the influence of organizational compatibility on green supply chain management efforts to boost organizational performance through information technology capabilities. *J. Cleaner Prod.* 266 1–13.

[B99] Shahzad M. Qu Y. Javed S. A. Zafar A. U. Rehman S. U. (2020). Relation of environment sustainability to CSR and green innovation: A case of Pakistani manufacturing industry. *J. Cleaner Prod.* 253 119938 10.1016/j.jclepro.2019.119938

[B100] Siguaw J. A. Simpson P. M. Enz C. A. (2006). Conceptualizing innovation orientation: A framework for study and integration of innovation research. *J. Prod.Innov. Manage.* 23 556–574. 10.1111/j.1540-5885.2006.00224.x

[B101] Singh S. K. El-Kassar A.-N. (2018). Green innovation and organizational performance: The influence of big data and the moderating role of management commitment and HR practices. *Technol. Forecast. Social Change* 144 483–498.

[B102] Soewarno N. Tjahjadi B. Fithrianti F. (2019). Green innovation strategy and green innovation: the roles of green organizational identity and environmental organizational legitimacy. *Manage. Decis.* 57 3061–3078. 10.1108/md-05-2018-0563

[B103] Song M. Yang M. X. Zeng K. J. Feng W. (2020). Green knowledge sharing, stakeholder pressure, absorptive capacity, and green innovation: Evidence from Chinese manufacturing firms. *Bus. Strategy Environ.* 29 1517–1531. 10.1002/bse.2450

[B104] Song W. Yu H. (2018). Green innovation strategy and green innovation: The roles of green creativity and green organizational identity. *Corporate Social Responsibility Environ.Manage.* 25 135–150. 10.1002/csr.1445

[B105] Stock R. M. Zacharias N. A. (2011). Patterns and performance outcomes of innovation orientation. *J. Acad. Marketing Sci.* 39 870–888. 10.1007/s11747-010-0225-2

[B106] Sulkowski A. J. Edwards M. Freeman R. E. (2018). Shake your stakeholder: Firms leading engagement to cocreate sustainable value. *Organ. Environ.* 31 223–241. 10.1177/1086026617722129

[B107] Tang M. Walsh G. Lerner D. Fitza M. A. Li Q. (2018). Green innovation, managerial concern and firm performance: An empirical study. *Bus. Strategy Environ.* 27 39–51. 10.1002/bse.1981

[B108] Tirabeni L. De Bernardi P. Forliano C. Franco M. (2019). How can organisations and business models lead to a more sustainable society? A framework from a systematic review of the industry 4.0. *Sustainability* 11:6363 10.3390/su11226363

[B109] Ullah M. (2017). Integrating environmental sustainability into human resource management: a comprehensive review on green human resource management. *Maghreb Rev. Eco. Manage.* 423 1–17. 10.4324/9781315768953-1

[B110] Umrani W. A. Kura K. M. Ahmed U. (2018). Corporate entrepreneurship and business performance: the moderating role of organizational culture in selected banks in pakistan. *PSU Res. Rev.* 2 59–80. 10.1108/prr-12-2016-0011

[B111] Venkatraman N. Ramanujam V. (1986). Measurement of business performance in strategy research: a comparison of approaches. *Acad. Manage. Rev.* 11 801–814. 10.2307/258398

[B112] Wagner M. (2007). On the relationship between environmental management, environmental innovation and patenting: evidence from german manufacturing firms. *Res. Policy* 36 1587–1602. 10.1016/j.respol.2007.08.004

[B113] Wang S.-H. Song M.-L. (2014). Review of hidden carbon emissions, trade, and labor income share in china, 2001–2011. *Energy Policy* 74 395–405. 10.1016/j.enpol.2014.08.038

[B114] Weng H.-H. R. Chen J.-S. Chen P.-C. (2015). Effects of green innovation on environmental and corporate performance: a stakeholder perspective. *Sustainability* 7 4997–5026. 10.3390/su7054997

[B115] Xie X. Huo J. Zou H. (2019). Green process innovation, green product innovation, and corporate financial performance: a content analysis method. *J. Bus. Res.* 101 697–706. 10.1016/j.jbusres.2019.01.010

[B116] Xie X. Zhu Q. Wang R. (2019). Turning green subsidies into sustainability: how green process innovation improves firms’ green image. *Bus. Strategy Environ.* 28 1416–1433. 10.1002/bse.2323

[B117] Yakubu O. (2017). Addressing environmental health problems in ogoniland through implementation of united nations environment program recommendations: environmental management strategies. *Environments* 4:28 10.3390/environments4020028

[B118] Yen Y.-X. Yen S.-Y. (2012). Top-management’s role in adopting green purchasing standards in high-tech industrial firms. *J. Bus. Res.* 65 951–959. 10.1016/j.jbusres.2011.05.002

[B119] Yu M. (2019). Impact of environmental regulation on green innovation practice of food enterprises: regulating effect of environmental awareness of different executives. *Rev. Facult. Agr. Univ. Zulia* 36 149696095.

[B120] Zeng S. Meng X. Zeng R. Tam C. M. Tam V. W. Jin T. (2011). How environmental management driving forces affect environmental and economic performance of SMEs: a study in the northern china district. *J. Cleaner Prod.* 19 1426–1437. 10.1016/j.jclepro.2011.05.002

[B121] Zhang H. He J. Shi X. Hong Q. Bao J. Xue S. (2020). Technology characteristics, stakeholder pressure, social influence, and green innovation: empirical evidence from chinese express companies. *Sustainability* 12;2891. 10.3390/su12072891

[B122] Zhang J. A. Walton S. (2017). Eco-innovation and business performance: the moderating effects of environmental orientation and resource commitment in green-oriented SME s. *R&d Manage.* 47 E26–E39.

[B123] Zhang J. Zhang X. Wang Q. Ma Z. (2019). “Relationship Between Institutional Pressures, Green Supply Chain Management Practices and Business Performance: An Empirical Research on Automobile Industry,” in *Paper presented at the International Conference on Management Science and Engineering Management.* San Francisco: ICMSEM.

[B124] Zhou K. Z. Gao G. Y. Yang Z. Zhou N. (2005). Developing strategic orientation in china: antecedents and consequences of market and innovation orientations. *J. Bus. Res.* 58 1049–1058. 10.1016/j.jbusres.2004.02.003

[B125] Zhu Q. Sarkis J. (2004). Relationships between operational practices and performance among early adopters of green supply chain management practices in chinese manufacturing enterprises. *J. Oper. Manage.* 22 265–289. 10.1016/j.jom.2004.01.005

[B126] Zhu Q. Feng Y. Choi S.-B. (2017). The role of customer relational governance in environmental and economic performance improvement through green supply chain management. *J. Cleaner Prod.* 155 46–53. 10.1016/j.jclepro.2016.02.124

[B127] Zhu Q. Geng Y. Fujita T. Hashimoto S. (2010). Green supply chain management in leading manufacturers: case studies in japanese large companies. *Manage. Res. Rev.* 33 380–392. 10.1108/01409171011030471

[B128] Zhu Q. Sarkis J. Cordeiro J. J. Lai K.-H. (2008). Firm-level correlates of emergent green supply chain management practices in the chinese context. *Omega* 36 577–591.

